# Supplementary figures and images for: Prediction of serious complications in patients with pulmonary thromboembolism and solid cancer: Validation of the EPIPHANY Index in a prospective cohort of patients from the PERSEO study
Source: PLoS One. 2023 May 9;18(5):e0266305. doi: 10.1371/journal.pone.0266305 (PMC10168567; doi:10.1371/journal.pone.0266305)

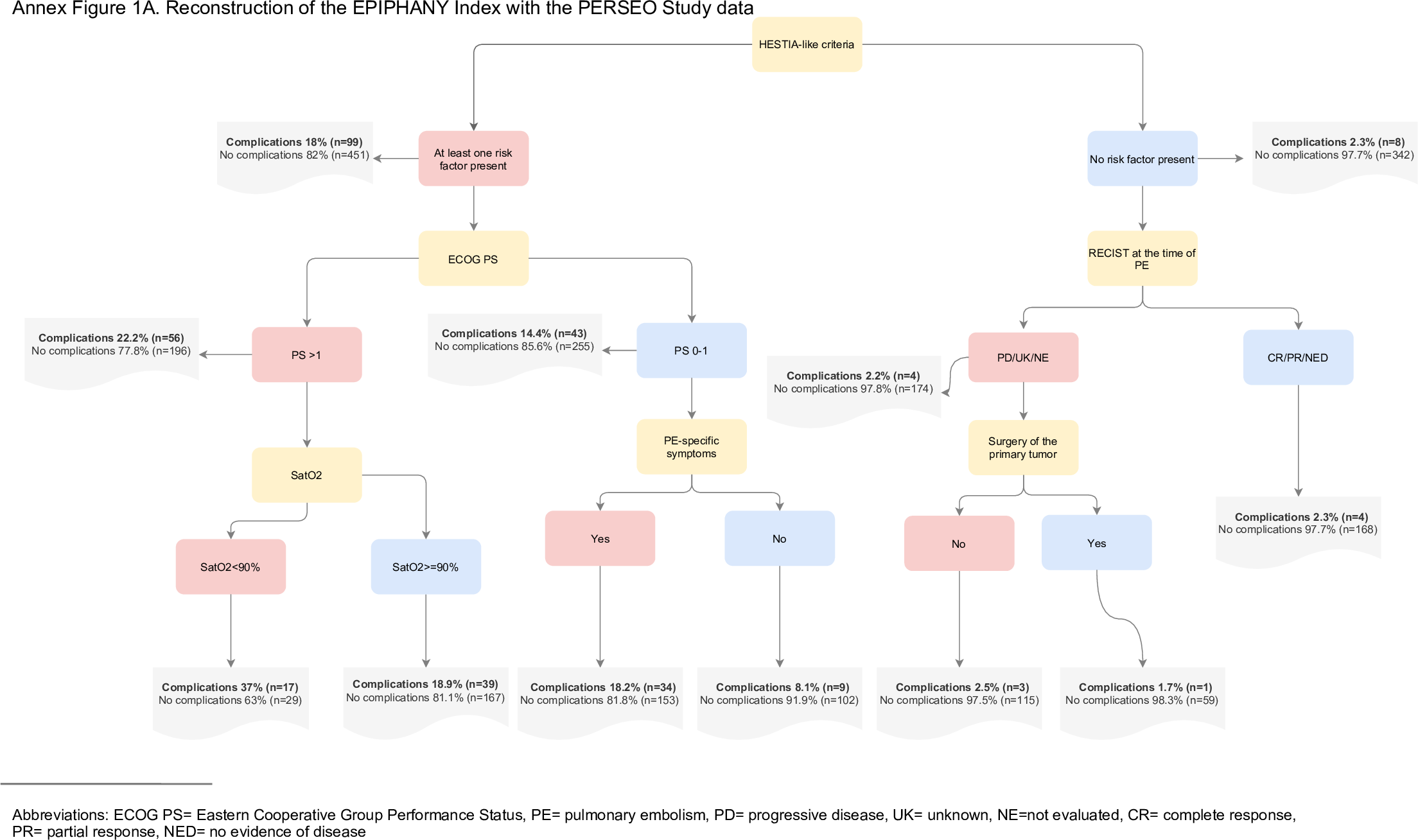

Supplement: S1 Fig — (TIF) [file pone.0266305.s002.tif]

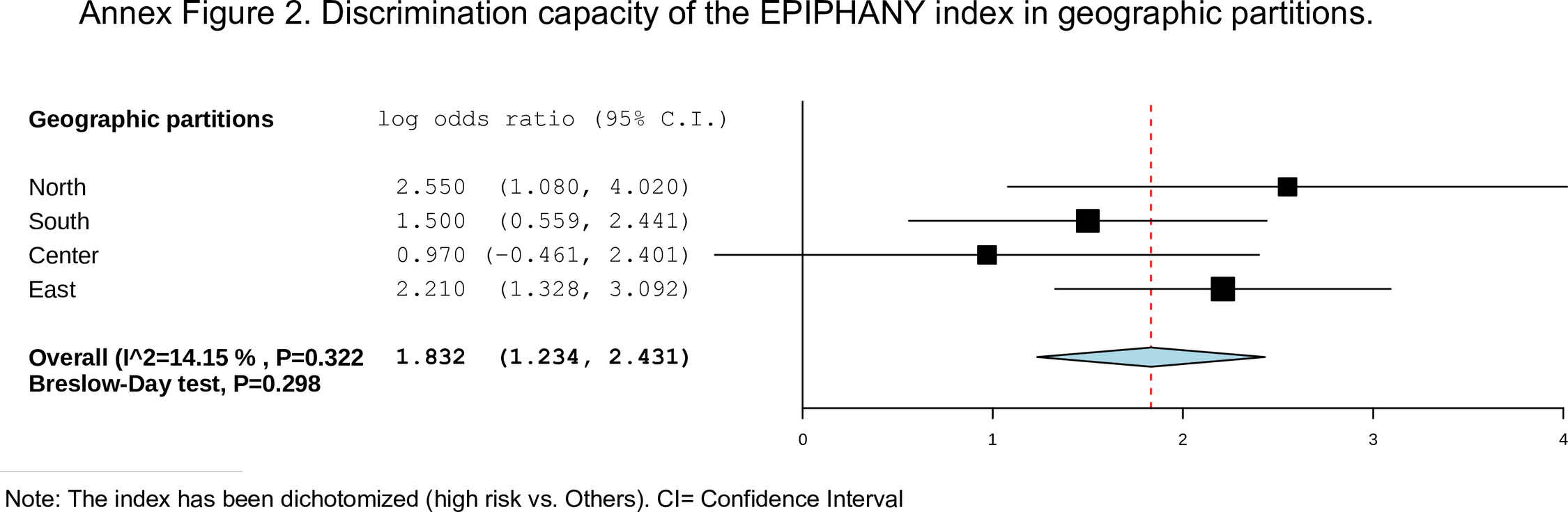

Supplement: S2 Fig — (TIF) [file pone.0266305.s003.tif]

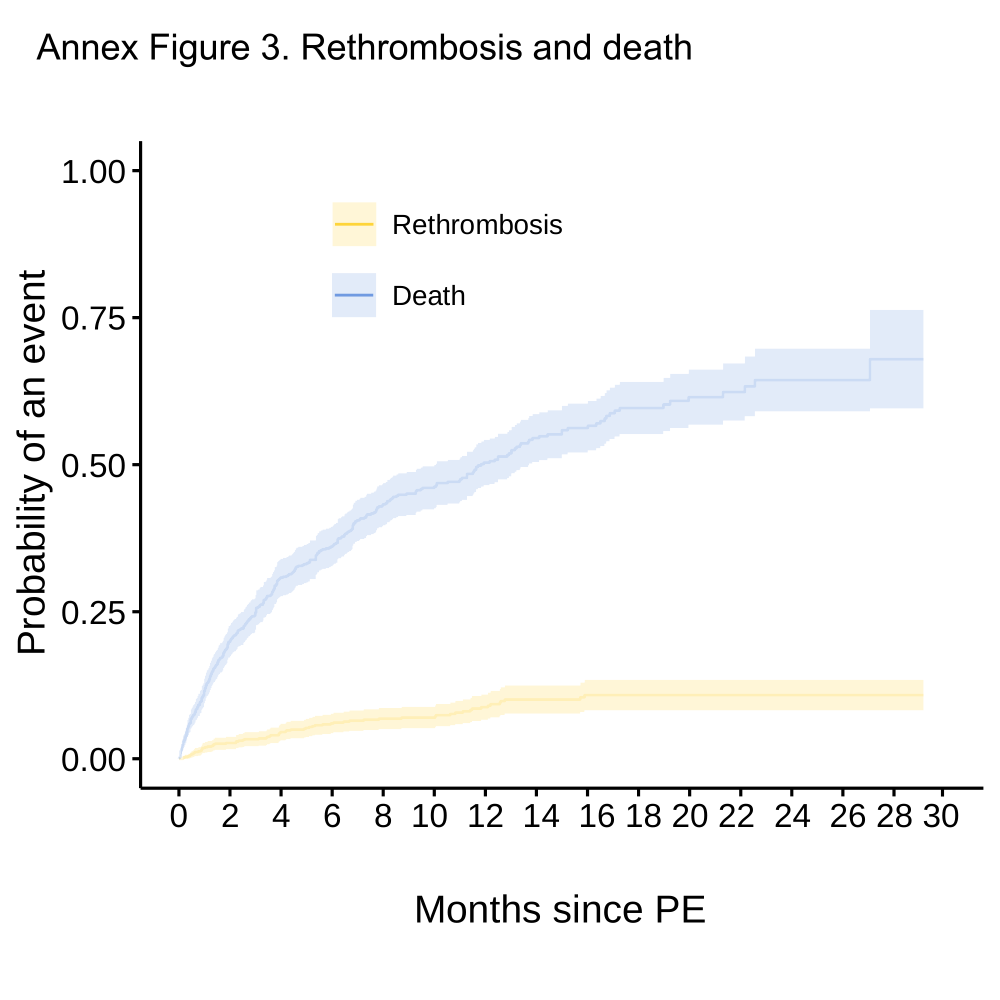

Supplement: S3 Fig — (TIFF) [file pone.0266305.s004.tiff]

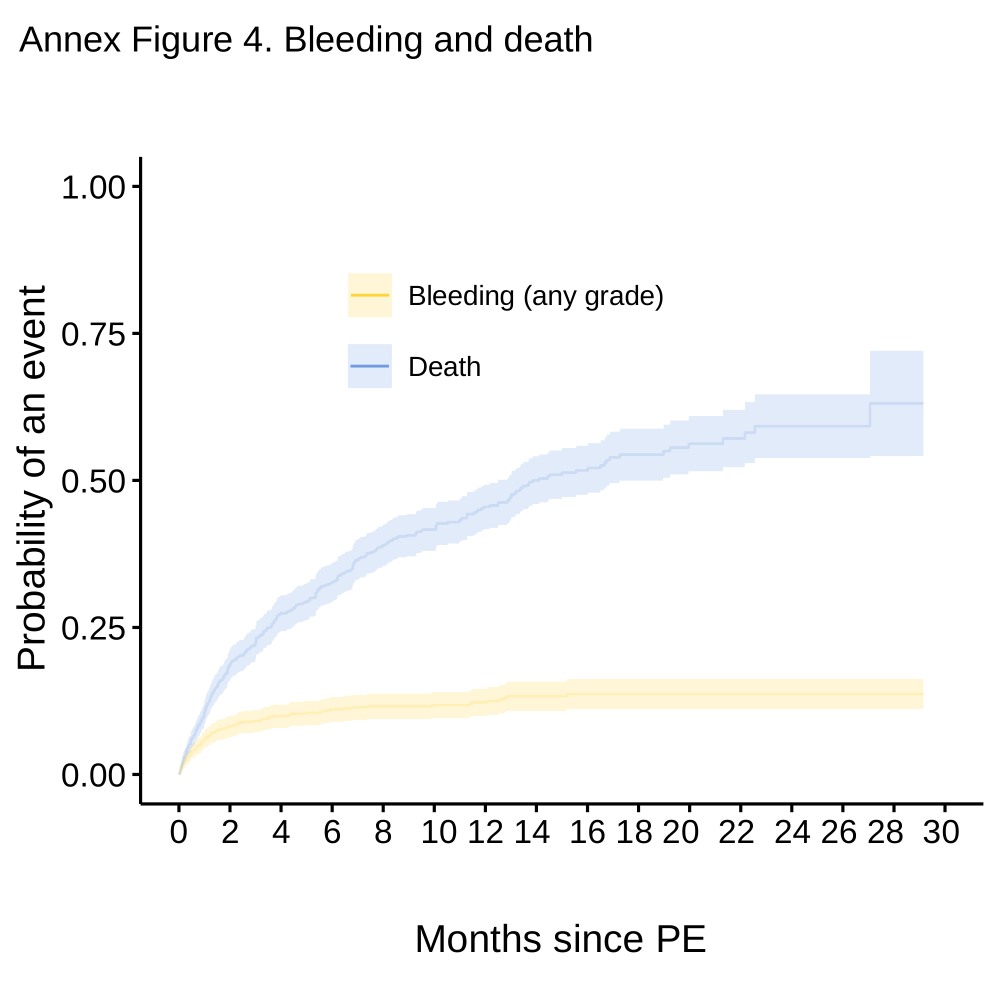

Supplement: S4 Fig — (TIFF) [file pone.0266305.s005.tiff]

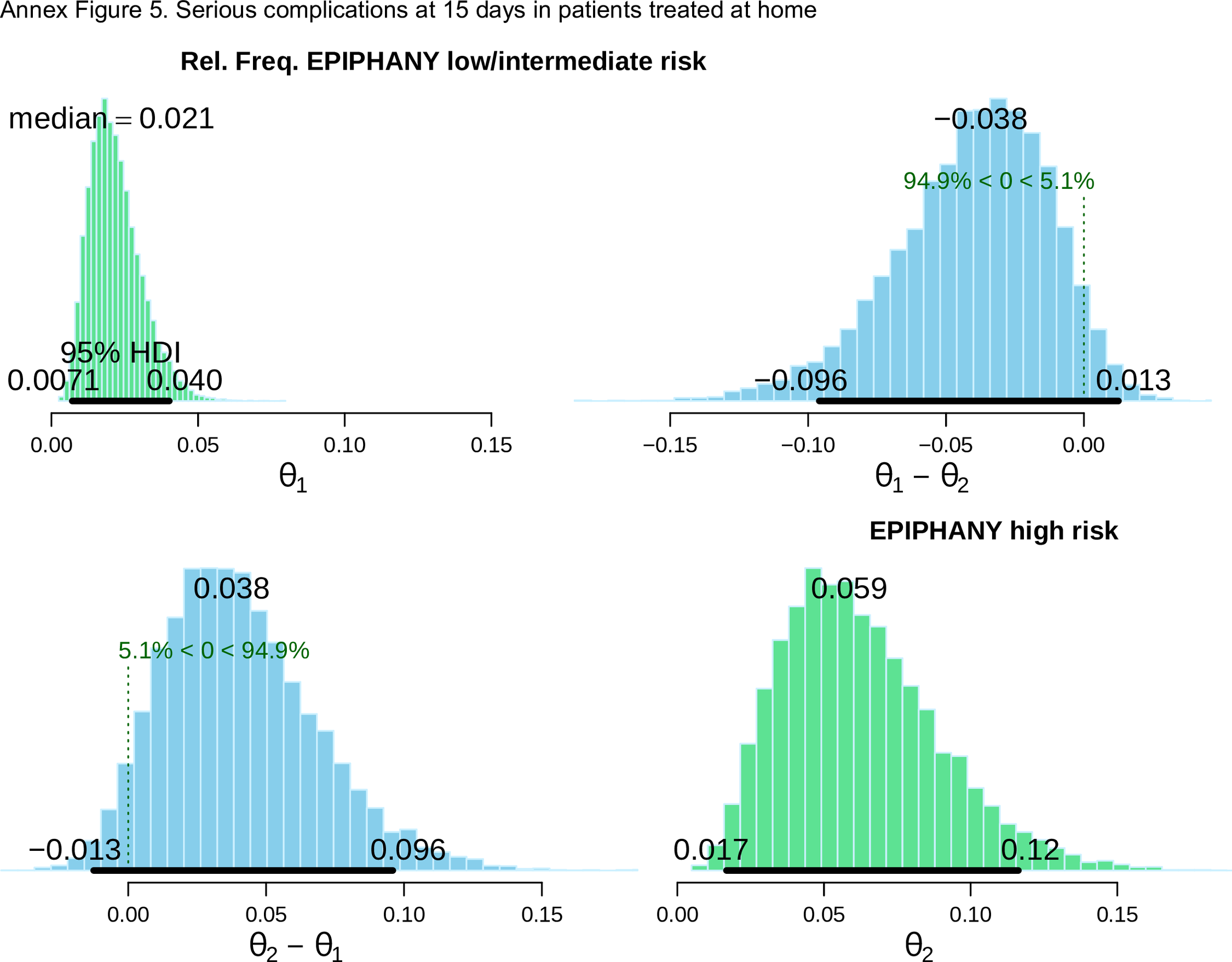

Supplement: S5 Fig — (TIF) [file pone.0266305.s006.tif]
