# Supplementary material for: Prediction of serious complications in patients with pulmonary thromboembolism and solid cancer: Validation of the EPIPHANY Index in a prospective cohort of patients from the PERSEO study
Source: PLoS One. 2023 May 9;18(5):e0266305. doi: 10.1371/journal.pone.0266305 (PMC10168567; doi:10.1371/journal.pone.0266305)
Supplement: S1 Table — (DOCX) [file pone.0266305.s007.docx]

**Annex table 1A. Baseline characteristics of the sample stratified by the laterality of pulmonary embolism.**

|  |  | Unilateral (n = 502) | Bilateral (n = 398) |
| --- | --- | --- | --- |
| Sex, male |  | 285 (56.9) | 232 (58.3) |
| Age, median (range) |  | 67 (25 – 92) | 65 (21-94) |
| ECOG-PS | 0 | 74 (14.7) | 62 (15.6) |
|  | 1 | 256 (51) | 190 (47.7) |
|  | 2 | 124 (24.7) | 105 (26.4) |
|  | 3 | 45 (9) | 37 (9.3) |
|  | 4 | 3 (0.6) | 4 (1) |
| Most common tumors | Lung | 151 (30.1) | 86 (21.6) |
|  | Colorrectal | 87 (17.3) | 81 (20.4) |
|  | Breast | 46 (9.2) | 27 (6.8) |
|  | Pancreatic | 38 (7.6) | 26 (6.5) |
|  | Bladder | 19 (3.8) | 28 (7) |
|  | Stomach | 26 (5.2) | 19 (4.8) |
|  | Ovarian | 22 (4.4) | 18 (4.5) |
|  | Central Nervous System | 11 (2.2) | 20 (5) |
|  | Endometrial | 16 (3.2) | 9 (2.3) |
| TNM classification, stage IV |  | 408 (81.3) | 297 (74.6) |
| RECIST al PE diagnosis | Not evaluable | 198 (39.4) | 220 (55.3) |
|  | Progression | 122 (24.3) | 82 (20.6) |
|  | Stable disease | 92 (18.3) | 52 (13.1) |
|  | Partial response | 62 (12.4) | 32 (8) |
|  | Complete response/NED | 28 (5.6) | 12 (3) |
| Comorbidities | Chronic cardiovascular disease | 60 (12) | 30 (7.5) |
|  | COPD | 54 (10.8) | 25 (6.3) |
|  | Chronic kidney disease | 19 (3.8) | 15 (3.8) |
|  | Chronic liver disease | 7 (1.4) | 6 (1.5) |
|  | Major surgery in the previous 3 months | 30 (6) | 20 (5) |
| Smoking status | Non-smoker | 98 (19.6) | 62 (15.6) |
|  | Ex-smoker | 17 (3.5) | 14 (3.4) |
|  | Active smoker | 163 (32.5) | 149 (37.4) |
|  | Unknown | 223 (44.4) | 174 (43.6) |
| Previous thrombosis |  | 79 (15.7) | 60 (15.1) |
| Anticoagulation therapy at diagnosis of PE |  | 66 (13.1) | 44 (11.1) |
| Anti-aggregation therapy at diagnosis of PE |  | 43 (8.6) | 28 (7) |
| PE diagnosis technique | Angio-CT | 131 (26.1) | 170 (42.7) |
|  | Ventilation/perfusion scintigraphy | 8 (1.6) | 10 (2.5) |
|  | CT for response assessment | 275 (54.8) | 162 (40.7) |
|  | CT for other reasons | 88 (17.5) | 56 (14.1) |
| Type of care | Outpatient | 226 (45) | 125 (31.4) |
|  | Inpatient | 276 (55) | 273 (68.6) |

*Abbreviations: COPD, Chronic Obstructive Pulmonary Disease; CT, computed tomography ECOG-PS, Eastern Cooperative Oncology Group; NED, No Evidence of Disease; PE, Pulmonary Embolism; RECIST, Response Evaluation Criteria In Solid Tumors.*

**Annex table 1B. Baseline characteristics of the sample stratified by PE location**

|  |  | Central  (n = 209) | Peripheral  (n =333) | Central and peripheral  (n = 358) |
| --- | --- | --- | --- | --- |
| Sex, male |  | 136 (65.1) | 185 (55.6) | 196 (54.7) |
| Age, median (range) |  | 67 (21 – 94) | 66 (25 – 92) | 66 (25 – 93) |
| ECOG-PS | 0 | 30 (14.4) | 53 (15.9) | 53 (14.8) |
|  | 1 | 106 (50.7) | 163 (48.9) | 177 (49.4) |
|  | 2 | 50 (23.9) | 83 (25) | 96 (26.8) |
|  | 3 | 20 (9.6) | 31 (9.3) | 31 (8.7) |
|  | 4 | 3 (1.4) | 3 (0.9) | 1 (0.3) |
| Most common tumors | Lung | 47 (22.5) | 94 (28.2) | 96 (26.8) |
|  | Colorrectal | 47 (22.5) | 49 (14.7) | 72 (20.1) |
|  | Breast | 15 (7.2) | 28 (8.4) | 30 (8.4) |
|  | Pancreatic | 19 (9.1) | 25 (7.5) | 20 (5.6) |
|  | Bladder | 14 (6.7) | 15 (4.5) | 18 (5) |
|  | Stomach | 13 (6.2) | 19 (5.7) | 13 (3.6) |
|  | Ovarian | 9 (4.3) | 16 (4.8) | 15 (4.2) |
|  | Central Nervous System | 10 (4.8) | 7 (2.1) | 14 (3.9) |
|  | Endometrial | 1 (0.5) | 10 (3) | 14 (3.9) |
| TNM classification, stage IV |  | 164 (78.5) | 263 (79) | 278 (77.7) |
| RECIST al PE diagnosis | Not evaluable | 80 (38.3) | 162 (48.6) | 176 (49.1) |
|  | Progression | 61 (29.2) | 69 (20.7) | 74 (20.7) |
|  | Stable disease | 38 (18.2) | 46 (13.8) | 60 (16.8) |
|  | Partial response | 26 (12.4) | 35 (10.5) | 33 (9.2) |
|  | Complete response/NED | 4 (1.9) | 21 (6.4) | 15 (4.2) |
| Comorbidities | Chronic cardiovascular disease | 24 (11.5) | 33 (9.9) | 33 (9.2) |
|  | COPD | 18 (8.6) | 33 (9.9) | 28 (7.8) |
|  | Chronic kidney disease | 10 (4.8) | 10 (3) | 14 (3.9) |
|  | Chronic liver disease | 3 (1.4) | 7 (2.1) | 3 (0.8) |
|  | Major surgery in the previous 3 months | 8 (3.8) | 24 (7.2) | 18 (5) |
| Smoking status | Non-smoker | 32 (15.3) | 68 (20.4) | 60 (16.8) |
|  | Ex-smoker | 4 (1.9) | 10 (3) | 17 (4.7) |
|  | Active smoker | 71 (34) | 113 (34) | 128 (35.8) |
|  | Unknown | 102 (48.8) | 142 (42.6) | 153 (42.7) |
| Previous thrombosis |  | 29 (13.9) | 55 (16.5) | 55 (15.4) |
| Anticoagulation therapy at diagnosis of PE |  | 30 (14.4) | 39 (11.7) | 41 (11.5) |
| Anti-aggregation therapy at diagnosis of PE |  | 21 (10) | 28 (8.4) | 22 (6.1) |
| PE diagnosis technique | Angio-CT | 48 (23) | 113 (33.9) | 140 (39.1) |
|  | Ventilation/perfusion scintigraphy | 3 (1.4) | 9 (2.7) | 6 (1.7) |
|  | CT for response assessment | 124 (59.3) | 156 (46.8) | 157 (43.9) |
|  | CT for other reasons | 34 (16.3) | 55 (16.6) | 55 (15.3) |
| Type of care | Outpatient | 95 (45.5) | 142 (42.6) | 114 (31.8) |
|  | Inpatient | 114 (54.5) | 191 (57.4) | 244 (68.2) |

*Abbreviations: COPD, Chronic Obstructive Pulmonary Disease; CT, computed tomography ECOG-PS, Eastern Cooperative Oncology Group; NED, No Evidence of Disease; PE, Pulmonary Embolism; RECIST, Response Evaluation Criteria In Solid Tumors.*

**Annex table 1C. Baseline characteristics of the sample stratified by presentation**

|  |  | Suspected  (n = 319) | Unsuspected, symptomatic  (n = 144) | Unsuspected, asymptomatic  (n = 437) |
| --- | --- | --- | --- | --- |
| Sex, male |  | 170 (53.3) | 88 (61.1) | 259 (59.3) |
| Age, median (range) |  | 65 (21-92) | 67 (38-93) | 67 (25-94) |
| ECOG-PS | 0 | 33 (10.3) | 13 (9) | 90 (20.6) |
|  | 1 | 133 (41.7) | 76 (52.8) | 237 (54.2) |
|  | 2 | 99 (31) | 42 (29.2) | 88 (20.1) |
|  | 3 | 49 (15.4) | 12 (8.3) | 21 (4.8) |
|  | 4 | 5 (1.6) | 1 (0.7) | 1 (0.3) |
| Most common tumors | Lung | 101 (31.7) | 52 (36.1) | 84 (19.2) |
|  | Colorrectal | 43 (13.5) | 20 (13.9) | 105 (24.1) |
|  | Breast | 32 (10) | 10 (6.9) | 31 (7.1) |
|  | Pancreatic | 23 (7.2) | 10 (6.9) | 31 (7.1) |
|  | Bladder | 10 (3.1) | 9 (6.3) | 28 (6.4) |
|  | Stomach | 5 (1.6) | 5 (3.5) | 35 (8) |
|  | Ovarian | 13 (4.1) | 5 (3.5) | 22 (5) |
|  | Central Nervous System | 24 (7.5) | 3 (2.1) | 4 (0.9) |
|  | Endometrial | 6 (1.9) | 5 (3.5) | 14 (3.2) |
| TNM classification, stage IV |  | 236 (74) | 117 (81.3) | 352 (80.5) |
| RECIST al PE diagnosis | Not evaluable | 258 (80.9) | 59 (41) | 101 (23.1) |
|  | Progression | 38 (11.9) | 44 (30.6) | 122 (27.9) |
|  | Stable disease | 18 (5.6) | 23 (16) | 103 (23.6) |
|  | Partial response | 4 (1.3) | 14 (9.7) | 76 (17.4) |
|  | Complete response/NED | 1 (0.3) | 4 (2.7) | 35 (8) |
| Comorbidities | Chronic cardiovascular disease | 26 (8.2) | 20 (13.9) | 44 (2.2) |
|  | COPD | 32 (10) | 12 (8.3) | 35 (8) |
|  | Chronic kidney disease | 7 (2.2) | 9 (6.3) | 18 (4.1) |
|  | Chronic liver disease | 4 (1.3) | 2 (1.4) | 7 (1.6) |
|  | Major surgery in the previous 3 months | 18 (5.6) | 6 (4.2) | 26 (5.9) |
| Smoking status | Non-smoker | 51 (16) | 38 (26.4) | 71 (16.3) |
|  | Ex-smoker | 7 (2.2) | 5 (3.5) | 19 (4.3) |
|  | Active smoker | 118 (37) | 51 (35.4) | 143 (32.7) |
|  | Unknown | 143 (44.8) | 50 (34.7) | 204 (46.7) |
| Previous thrombosis |  | 47 (14.7) | 17 (11.8) | 75 (17.2) |
| Anticoagulation therapy at diagnosis of PE |  | 39 (12.2) | 15 (10.4) | 56 (12.8) |
| Anti-aggregation therapy at diagnosis of PE |  | 18 (5.6) | 18 (12.5) | 35 (8) |
| PE diagnosis technique | Angio-CT | 293 (91.9) | 8 (5.5) | 0 (0) |
|  | Ventilation/perfusion scintigraphy | 18 (5.6) | 0 (0) | 0 (0) |
|  | CT for response assessment | 0 (0) | 78 (54.2) | 359 (82.1) |
|  | CT for other reasons | 8 (2.5) | 58 (40.3) | 78 (17.9) |
| Type of care | Outpatient | 39 (12.2) | 38 (26.4) | 274 (62.7) |
|  | Inpatient | 280 (87.8) | 106 (73.6) | 163 (37.3) |

*Abbreviations: COPD, Chronic Obstructive Pulmonary Disease; CT, computed tomography ECOG-PS, Eastern Cooperative Oncology Group; NED, No Evidence of Disease; PE, Pulmonary Embolism; RECIST, Response Evaluation Criteria In Solid Tumors.*
