# Supplementary material for: Prediction of serious complications in patients with pulmonary thromboembolism and solid cancer: Validation of the EPIPHANY Index in a prospective cohort of patients from the PERSEO study
Source: PLoS One. 2023 May 9;18(5):e0266305. doi: 10.1371/journal.pone.0266305 (PMC10168567; doi:10.1371/journal.pone.0266305)
Supplement: S2 Table — (DOCX) [file pone.0266305.s008.docx]

**Annex Table 2. 30-day causes of death**

| **Causes of death** | **Patients who died during the study follow-up**  **N=434 (100%)** | **Patients deceased in the first 30 days post-diagnosis of PE**  **N=104 (100%)** |
| --- | --- | --- |
| **Complications of PE** | 10 (2.3) | 9 (8.7) |
| **Cancer progression** | 322 (74.2) | 45 (43.3) |
| **Mixed (PE complications and cancer progression)** | 52 (12) | 35 (33.7) |
| **Rethrombosis** | 4 (0.9) | 0 |
| **Bleeding** | 19 (4.4) | 7 (6.7) |
| **Infection** | 19 (4.4) | 6 (5,8) |
| **Other causes of death** | 8 (1.8) | 2 (1.9) |

Abbreviations: PE, Pulmonary Embolism.
