# Supplementary material for: Prediction of serious complications in patients with pulmonary thromboembolism and solid cancer: Validation of the EPIPHANY Index in a prospective cohort of patients from the PERSEO study
Source: PLoS One. 2023 May 9;18(5):e0266305. doi: 10.1371/journal.pone.0266305 (PMC10168567; doi:10.1371/journal.pone.0266305)
Supplement: S3 Table — (DOCX) [file pone.0266305.s009.docx]

**Annex Table 3.** **Comparison of performance of various prediction models (serious complications)**

|  | **EPIPHANY, % (CI 95%)**  **High/intermediate vs low risk** | | **EPIPHANY, % (CI 95%)**  **High vs /intermediate/low risk** | | **HESTIA, % (CI 95%)** | | **RIETE,**  **% (CI 95%)** | **PESI,**  **% (CI 95%)** | **Simplified PESI,**  **% (CI 95%)** | **Spanish score, % (CI 95%)** | **Geneva score,**  **% (CI 95%)** |
| --- | --- | --- | --- | --- | --- | --- | --- | --- | --- | --- | --- |
|  | **S-PE + I-PE** | **S-PE** | **S-PE + I-PE** | **S-PE** | **S-PE + I-PE** | **S-PE** | **S-PE** | **S-PE** | **S-PE** | **S-PE** | **S-PE** |
| **Sens** | 95.3  (89.5-97.9) | 98.4  (91.9-99.7) | 84.1  (75.7-90.4) | 93.9  (85.2-98.3) | 92.5  (85.3-96.4) | 96.9  (88.5-99.4) | 83.3  (72.5-90.4) | 93.9  (85.4-97.6) | 100 | 93.9  (85.4-97.6) | 69.7  (57.7-79.4) |
| **Spec** | 28.6  (25.5-31.8) | 3.9  (2.1-7.1) | 55.9  (52.4-59.4) | 14.6  (10.5-19.5) | 43.1  (39.6-46.6) | 9.09  (5.97-13.5) | 21.74  (17.1-27.2) | 6.7  (4.24-10.5) | 0 | 8.3  (5.4-12.3) | 48.6  (42.5-54.7) |
| **PPV** | 15.2  (12.7-18.2) | 21.1  (16.9 – 22.2) | 20.5  (16.8-24.5) | 22.3  (17.5-27.6) | 18.0  (14.9-21.5) | 21.7  (17.2-27.0) | 21.7  (17.1-27.2) | 20.8  (16.5-25.7) | 22.8  (18.4-27.9) | 21.0  (16.8-26.1) | 26.1  (20.2-33.0) |
| **NPV** | 97.8  (94.3-98.9) | 90.9  (62.2-98.3) | 96.3  (94.1-97.8) | 90.2  (76.8-97.2) | 97.7  (95.3-98.9) | 92  (72.5-98.6) | 83.3  (72.5- 90.4) | 80.95%  (60.0-92.3) | NC | 84  (63.0-94.7) | 86.0  (79.3-90.7) |
| **PLR** | 1.34 | 1.03 | 1.91 | 1.10 | 1.62 | 1.06 | 1.06 | 1.01 | 1 | 1.02 | 1.36 |
| **NLR** | 0.16 | 0.38 | 0.28 | 0.41 | 0.17 | 0.33 | 0.77 | 0.9 | NC | 0.73 | 0.62 |

Notes: Response variable: 15-day serious complications. Abbreviations: Spec, specificity, NLR: negative likelihood ratio, PLR: positive likelihood ratio, Sens: sensitivity, I-PE: incidental pulmonary thromboembolism, S-PE: suspected pulmonary thromboembolism, NPV: negative predictive value, PPV: positive predictive value, NC: not computable, CI: confidence interval.
