# Supplementary material for: Prediction of serious complications in patients with pulmonary thromboembolism and solid cancer: Validation of the EPIPHANY Index in a prospective cohort of patients from the PERSEO study
Source: PLoS One. 2023 May 9;18(5):e0266305. doi: 10.1371/journal.pone.0266305 (PMC10168567; doi:10.1371/journal.pone.0266305)
Supplement: S4 Table — (DOCX) [file pone.0266305.s010.docx]

**Annex Table 4.** **Comparison of performance of various prediction models (30-day mortality)**

|  | **EPIPHANY. % (95% CI)**  **High/intermediate vs low risk** | | **EPIPHANY. % (95% CI)**  **High vs /intermediate/low risk** | | **HESTIA. % (95% CI)** | | **RIETE.**  **% (95% CI)** | **PESI.**  **% (95% CI)** | **Simplified PESI.**  **% (95% CI)** | **Spanish score. % (95% CI)** | **Geneva score.**  **% (95% CI)** |
| --- | --- | --- | --- | --- | --- | --- | --- | --- | --- | --- | --- |
|  | **S-PE + I-PE** | **S-PE** | **S-PE + I-PE** | **S-PE** | **S-PE + I-PE** | **S-PE** | **S-PE** | **S-PE** | **S-PE** | **S-PE** | **S-PE** |
| **Sens** | 98.0  (92.5-99.6) | 100 | 79.8  (70.8-87.0) | 96.3  (87.4-99.5) | 87.5  (79.9-92.9) | 98.1  (89-99.9) | 98.1  (89-99.9) | 94.5  (83.9-98.5) | 100 | 100 | 74.5  (60.7-84.9) |
| **Spec** | 28.8  (25.8-32.1) | 4.1  (2.3-7.3) | 55.2  (51.7-58.7) | 14.7  (10.7-19.6) | 42.3  (38.8-45.8) | 9.0  (6.0-13.3) | 24.6  (19.6-30.3) | 6.8  (4.2-10.7) | 0 | 9.4  (6.3-13.8) | 48.8  (42.7-55.0) |
| **PPV** | 15.2  (12.7-18.2) | 17.8  (13.9-22.5) | 18.9  (15.3-22.8) | 19.0  (14.6-24.1) | 16.5  (13.5-19.9) | 18.3  (14.2-23.3) | 21.3  (16.5-27.0) | 17.4  (13.4-22.3) | 17.2  (13.3-21.9) | 18.7  (14.5-23.7) | 23.3  (17.4-30.3) |
| **NPV** | 99.1  (96.9-99.7) | 100 | 95.4  (93.1-97-1) | 95.1  (83.4-99.4) | 96.2  (93.5-97.9) | 96  (77.6-99.7) | 98.4%  (90.7-99.9) | 85.7  (62.6-96.2) | NC | 100 | 90.2  (83.8-94.3) |
| **PLR** | 1.38 | 1.04 | 1.78 | 1.13 | 1.52 | 1.08 | 1.3 | 1.01 | 1 | 1.1 | 1.45 |
| **NLR** | 0.07 | 0 | 0.36 | 0.24 | 0.29 | 0.2 | 0.07 | 0.8 | NC | 0 | 0.52 |

Notes: Response variable: 30-day mortality. Abbreviations: NLR: negative likelihood ratio, PLR: positive likelihood ratio, Sens: sensitivity, Spec, specificity, I-PE: incidental pulmonary thromboembolism, S-PE: suspected pulmonary thromboembolism, NPV: negative predictive value, PPV: positive predictive value, NC, not computable, CI: confidence interval.
