# Supplementary material for: Prediction of serious complications in patients with pulmonary thromboembolism and solid cancer: Validation of the EPIPHANY Index in a prospective cohort of patients from the PERSEO study
Source: PLoS One. 2023 May 9;18(5):e0266305. doi: 10.1371/journal.pone.0266305 (PMC10168567; doi:10.1371/journal.pone.0266305)
Supplement: S5 Table — (DOCX) [file pone.0266305.s011.docx]

**Annex Table 5. Multivariate analysis: predictors of serious complications**

| **Multivariable analysis** | **Beta coefficient** | **OR** | **95% CI** | **p-value** |
| --- | --- | --- | --- | --- |
| **Presence of >1 modified Hestia criteria** | 1.29 | 3.663 | 1.568 - 8.42 | 0.003 |
| **ECOG-PS 2-4** | 0.469 | 1.599 | 1.037 - 2.464 | 0.034 |
| **SpO2 <90%** | 0.646 | 1.908 | 1.114 - 3.267 | 0.019 |
| **Presence of specific symptoms of PE** | 0.643 | 1.902 | 1.119 - 3.234 | 0.018 |
| **Progressing or non-classifiable cancer according to RECIST** | 0.751 | 2.119 | 1.102 - 4.077 | 0.024 |
| **Absence of resection of the primary tumor** | 0.582 | 1.79 | 0.953 - 3.362 | 0.07 |

Abbreviations: ECOG-PS, Eastern Cooperative Oncology Group – Performance Status; SpO2, oxygen saturation levels; RECIST, Response Evaluation Criteria In Solid Tumors; PE, Pulmonary Embolism; OR, Odds Ratio; CI, Confidence Interval. A binary logistic regression model was used. The full model has an AUC of 0.710 (95% CI, 0.673-0.746) vs 0.678 (95% CI, 0.647-0.708) for the modified Hestia criteria (DeLong test, p-value=0.005).
