# Supplementary material for: Prediction of serious complications in patients with pulmonary thromboembolism and solid cancer: Validation of the EPIPHANY Index in a prospective cohort of patients from the PERSEO study
Source: PLoS One. 2023 May 9;18(5):e0266305. doi: 10.1371/journal.pone.0266305 (PMC10168567; doi:10.1371/journal.pone.0266305)
Supplement: S6 Table — (DOCX) [file pone.0266305.s012.docx]

**Annex Table 6.** Serious complications at 15 days based on type of PE & location of management.

| **Scale** | **Hospitalized,**  **% (95% HDI)** | **Hospitalized, n/N** | **Ambulatory,**  **% (95% HDI)** | **Ambulatory,**  **n/N** | **Suspected,**  **% (95% HDI)** | **Suspected, n/N** | **Unsuspected, % (95% HDI)** | **Unsuspected, asymptomatic, n/N** | **Unsuspected, symptomatic,**  **% (95% HDI)** | **Unsuspected, symptomatic, n/N** |
| --- | --- | --- | --- | --- | --- | --- | --- | --- | --- | --- |
| **Low-risk Epiphany** | 1.6 (0.1-6.5) | 1/68 | 2.5 (0.8-5.7) | 4/159 | 10.5 (1.0-36.5) | 1/11 | 2.0 (0.6-4.5) | 4/206 | 0.0 (0-0.1) | 0/15 |
| **Intermediate-risk Epiphany** | 9.3 (4.9-15.5) | 11/119 | 1.1 (0.1-4.2) | 1/110 | 10.5 (2.8-24.8) | 3/30 | 5.2 (2.6-9.2) | 9/173 | 0.0 (0-0.0) | 0/26 |
| **High-risk Epiphany** | 23.9 (19.7-28.5) | 86/360 | 5.3 (1.7-11.8) | 4/78 | 22.3 (17.6-27.5) | 62/278 | 10.5 (4.4-19.8) | 6/58 | 21.4 (14.4-29.9) | 22/103 |
| **Low-risk HESTIA** | 2.6 (0.7-6.7) | 3/119 | 2.2 (0.8-4.6) | 5/231 | 8.5 (1.6-23.2) | 2/25 | 2.0 (0.8-4.1) | 6/301 | 0.0 (0-0.1) | 0/24 |
| **High-risk HESTIA** | 22.1 (18.3-26.1) | 95/429 | 3.4 (1.1-7.6) | 4/120 | 21.8 (17.4-26.7) | 64/294 | 9.6 (5.4-15.4) | 13/136 | 18.4 (12.2-25.9) | 22/120 |

Abbreviations: HDI=highest density interval, n= events, N= sample size.
