# Supplementary material for: Prediction of serious complications in patients with pulmonary thromboembolism and solid cancer: Validation of the EPIPHANY Index in a prospective cohort of patients from the PERSEO study
Source: PLoS One. 2023 May 9;18(5):e0266305. doi: 10.1371/journal.pone.0266305 (PMC10168567; doi:10.1371/journal.pone.0266305)
Supplement: S7 Table — (DOCX) [file pone.0266305.s013.docx]

**Annex Table 7. Distribution of risk classes and 30-day mortality by EPIHANY index/ modified HESTIA criteria and treatment site**

| **Scale** | **Total,**  **% (95% HDI)** | **Inpatient, % (95% HDI)** | **Outpatient, % (95% HDI)** | **Suspected,**  **% (95% HDI)** | **Unsuspected, asymptomatic; % (95% HDI)** | **Unsuspected, symptomatic;**  **% (95% HDI)** |
| --- | --- | --- | --- | --- | --- | --- |
| **Low-risk Epiphany** | 0.9 (0.1-2.6) | 3.1 (0.5-9.2) | 0.0 (0.0-0.0) | 0.0 (0.0-0.2) | 0.0 (0.0-0.1) | 13.9 (2.8-36.2) |
| **Intermediate-risk Epiphany** | 8.3 (5.2-12.3) | 9.3 (4.9-15.5) | 7.3 (3.4-13.1) | 7.2 (1.4-19.4) | 8.7 (5-2-13.5) | 8.0 (1.6-22.2) |
| **High-risk Epiphany** | 18.9 (15.4-22.9) | 21.9 (17.8-26.3) | 5.2 (1.7-11.7) | 19.0 (14.7-23.9) | 19.1 (10.5-30.4) | 18.5 (11.9-26.7) |
| **Low-risk HESTIA** | 3.7 (2.0-6.0) | 5.9 (2.6-11.3) | 2.6 (1.0-5.2) | 4.6 (0.4-17.6) | 3.3 (1.6-5.8) | 8.9 (1.8-24.2) |
| **High-risk HESTIA** | 16.5 (13.6-19.8) | 19.8 (16.2-23.6) | 5.0 (2.1-9.9) | 18.3 (14.1-23.0) | 11.8 (7.1-17.8) | 17.5 (11.5-24.8) |

Abbreviation: HDI, highest density interval.
