# Supplementary material for: Prediction of serious complications in patients with pulmonary thromboembolism and solid cancer: Validation of the EPIPHANY Index in a prospective cohort of patients from the PERSEO study
Source: PLoS One. 2023 May 9;18(5):e0266305. doi: 10.1371/journal.pone.0266305 (PMC10168567; doi:10.1371/journal.pone.0266305)
Supplement: S8 Table — (DOCX) [file pone.0266305.s014.docx]

| **HESTIA criteria at diagnosis of pulmonary embolism** | **All,**  **N=900 (%)** | **Outpatients,**  **N=351 (%)** | **Inpatients,**  **N=540 (%)** |
| --- | --- | --- | --- |
| **At least 1 HESTIA criteria present** | 550 (61.1) | 120 (34.2) | 430 (78.3) |
| **Sudden or progressive dyspnea** | 337 (37.4) | 47 (13.4) | 290 (52.8) |
| **Respiratory rate ≥30 bpm** | 23 (2.6) | 4 (1.1) | 19 (3.5) |
| **SpO2 (ambient air) <90%** | 89 (9.9) | 3 (0.9) | 86 (15.7) |
| **Systolic pressure <100 mmHg** | 80 (8.9) | 16 (4.6) | 64 (11.7) |
| **Heart rate >110** **bpm** | 151 (16.8) | 20 (5.7) | 131 (23.9) |
| **Platelet <50000 cells/mm3** | 12 (1.3) | 2 (0.6) | 10 (1.8) |
| **High bleeding risk** | 144 (16.0) | 55 (15.7) | 89 (16.2) |
| **Concomitant, clinically relevant bleeding** | 6 (0.7) | 1 (0.3) | 5 (0.9) |

**Annex Table 8. HESTIA criteria in patients cared for in hospital and at home**

Abbreviations: ≥30 bpm, breaths per minute; >110 bpm, beats per minute; SpO2, oxygen saturation levels.
