# Supplementary material for: Prediction of serious complications in patients with pulmonary thromboembolism and solid cancer: Validation of the EPIPHANY Index in a prospective cohort of patients from the PERSEO study
Source: PLoS One. 2023 May 9;18(5):e0266305. doi: 10.1371/journal.pone.0266305 (PMC10168567; doi:10.1371/journal.pone.0266305)
Supplement: Attachment — Submitted filename: response_reviewers.docx [file pone.0266305.s015.docx]

PONE-D-22-07801

Prediction of serious complications in patients with cancer and pulmonary thromboembolism: validation of the EPIPHANY Index in a prospective cohort of patients from the PERSEO Study

Dear Dr. Sánchez Cánovas,

Thank you for submitting your manuscript to PLOS ONE. After careful consideration, we feel that it has merit but does not fully meet PLOS ONE’s publication criteria as it currently stands. Therefore, we invite you to submit a revised version of the manuscript that addresses the points raised during the review process. [….] We look forward to receiving your revised manuscript.

Kind regards,

Roza Chaireti

Firstly, we would like to express our gratitude to the editors of PLOS ONE for their perseverance in finding reviewers for this manuscript and for considering it as a meritorious research. Next, we respond point by point to each of the queries and concerns raised during the review process, incorporating the changes into the manuscript.

Journal Requirements:

When submitting your revision, we need you to address these additional requirements.

1. Please ensure that your manuscript meets PLOS ONE's style requirements, including those for file naming. The PLOS ONE style templates can be found at

https://journals.plos.org/plosone/s/file?id=wjVg/PLOSOne_formatting_sample_main_body.pdf and

https://journals.plos.org/plosone/s/file?id=ba62/PLOSOne_formatting_sample_title_authors_affiliations.pdf

2. Your ethics statement should only appear in the Methods section of your manuscript. If your ethics statement is written in any section besides the Methods, please delete it from any other section.

Reviewers' comments:

[….]

Reviewer #1: In this study authors aimed to prospectively validate the EPIPHANY Index and Hestia criteria in a broad sample of patients with cancer, with a view to their possible appropriateness in decision making. With this purpose, authors made a prospective, multicenter study with nine hundred patients diagnosed with PE between 2017/2020 were recruited

The rate of serious complications at 15 days was 11.8% (9.8-14.1%). Of the EPIPHANY low-risk patients, 2.4% (0.8-4.6%) had serious complications, as did 5.5% (2.9-8.7%) of the moderate-risk participants and 21.0% (17.0-24.0%) of those with high-risk episodes.

The concept of the study is interesting although there are several limitations that author need to address:

Please, edit English and review the text to avoid misunderstandings.

Firstly, we would like to thank the reviewer for their critical reading of the manuscript, for pointing out possible weaknesses or inaccuracies, and for suggesting ways to improve the analysis.

In accordance with this reviewer's comment, we have re-read the text and made an effort to simplify and clarify the language as much as possible.

Abstract:

Introduction of the abstract: please, verify this sentence “This study has validated the EPIHANY Index”.

We have corrected the typographical error in the revised version of the manuscript. Thank you for bringing it to our attention.

Include month in the period of inclusion.

Following the reviewer's suggestion, we have added the recruitment dates to the revised version of the abstract.

This sentence in the abstract need to be supported with data: “The EPIPHANY Index correlated with overall survival”

As per the reviewer's recommendation, we have included additional information in the revised manuscript to support the comment on overall survival in the abstract. The revised version now includes the following: *The EPIPHANY Index was associated with overall survival (OS) in patients with different risk levels, with median OS of 16.5, 14.4, and 4.4 months for those at low, intermediate, and high risk, respectively.*

Introduction. More references should be included in the 30 day mortality secondary to PE to provide a global idea to readers. There are other studies with lower mortality at 30 days.

The purpose of highlighting the 30-day mortality rate at the beginning of the introduction is to underscore the prognostic impact of pulmonary embolism in patients with cancer. We agree with Reviewer #1 that there may be patient profiles with a more favorable prognosis. For instance, in the series by Font et al, the 30-day mortality was 18% and 3% in inpatients and outpatients, respectively, with most of the domiciliary-treated patients having incidental episodes. Nevertheless, the prognosis for cancer patients with PE is substantially worse than for the general population without cancer (as shown in studies such as Jiménez et al, JAMA 2010 ^[1]^). In the introduction of our manuscript, we aim to describe pulmonary embolism as one of the most severe complications in cancer patients, without delineating specific patient profiles. To support the 30-day mortality rate, we have added references to six international series reporting mortality rates ranging from 15% to 25% ^[2]^ ^[3]^ ^[4]^ ^[5]^ ^[6]^ ^[7]^. Given that the rest of the manuscript discusses the existence of patient profiles with varying risks, we believe that providing the average mortality rate in this section of the introduction is sufficient to quickly convey the importance of this issue to the reader.

The revised version of the manuscript now includes the following statement:

*Pulmonary embolism (PE) has classically been deemed one of the most common and dire complications in patients with cancer [1]. Studies show that the 30-day mortality rates for acute PE, which often requires intensive hospital management, range from 15% to 25% across different series [2–8].*

Methods section

- If investigators followed patients at 30 days, why they analyzed complications at 15 days?

We agree with Reviewer #1 that this point requires clarification. The EPIPHANY index is intended to assist in identifying eligible patients who can safely receive outpatient management, while reducing the risk of PE-specific complications. The model assumes that decisions regarding patients at the end of life would be based on other considerations, such as quality of life, within the palliative care framework, rather than solely relying on the risk-based score. Therefore, one notable difference between the EPIPHANY index and previous classifications is the proposal to use the probability of serious complications within 15 days as the primary endpoint, rather than all-cause 30-day mortality, which has typically been used in other studies (Aujesky et al, 2006; Jiménez et al, 2010; Kline et al, 2012; Den Exter et al, 2013). We acknowledge that mortality is a more robust outcome, and for that reason, it has been maintained as a secondary endpoint. However, after evaluating the performance of various prognostic scales in this situation, our group proposed in 2016 that the rate of complications at 15 days was possibly the most interesting outcome if the goal was outpatient management (Carmona-Bayonas et al, 2016)^[8]^. One argument for this proposal is that the appearance of serious complications in patients with PE treated as outpatients, far from medical supervision, can paradoxically turn low-risk patients into the most vulnerable, due to misclassification. Therefore, we wanted to increase sensitivity to detect early complications associated with PE. In contrast, our analysis showed that the probability of all-cause 30-day mortality did not necessarily affect decision-making regarding outpatient treatment in some subgroups, as the cause of death was rarely the PE itself, and often due to cancer progression (Den Exter et al, 2013; Carmona-Bayonas et al, 2016). In fact, patients in palliative care for advanced disease were those in whom it was even more important to avoid unnecessary hospitalizations at the end of their lives, and often scored as higher-risk patients. Therefore, using all-cause 30-day mortality as an endpoint led to proposing intensification of PE management (e.g., with fibrinolysis) in subjects at greater risk of early mortality due to cancer, who were less likely to benefit from this type of treatment. Had we employed all-cause 30-day mortality as the primary endpoint, as is typically done by other authors, more than half of the anticipated events would have been attributed to tumor progression rather than the direct impact of PE. For instance, when we examined the causes of death at 15, 30, and 90 days after PE within the original derivation series of the EPIPHANY index, cancer was the cause of death in 35%, 54%, and 65%, respectively ^[9]^. For this reason, our validation of the EPIPHANY model has remained true to the original study design, using the rate of complications at 15 days as the primary endpoint, and all-cause 30-day mortality as the secondary endpoint, which is the most classic outcome.

In the revised manuscript, we have provided a clarification that serious complications within 15 days were taken into consideration to perform the validation:

*In our model validation process, we selected serious complications within 15 days as the primary endpoint. This choice was made based on its better fit with the clinical scenario of managing pulmonary embolism (PE) in cancer patients as outpatients. This decision was previously supported in the literature [8,14].*

- Authors need to specify in which moment patients were recruited. At diagnosis? How long from diagnosis PE?

In accordance with the reviewer's recommendations, in the revised version we have included when patient recruitment was carried out. Since PERSEO is a prospective study that required the evaluation of acute clinical parameters, patient recruitment occurred on the same day of the diagnosis of PE in all cases. Clinicians were instructed to evaluate vital signs and specific symptoms of PE directly from the patients, during the acute phase. Consequently, we have introduced the following clarifying sentence in the materials and methods section:

*Patient recruitment was conducted during the acute phase of PE (within the first three hours) to enable the direct collection of vital signs and symptom data.*

- Objective: Secondary objective need to be clarified. Compare with “other models” what means? Due to it was a prospective study this objectives needed to be pre-specified. The other models need to be clear and named.

We agree with the reviewer that the comparison of the EPIPHANY index with other scores should be more clearly reflected. Therefore, we have specified which models have been evaluated, providing appropriate references in each case. The revised manuscript now specifies:

*The secondary objectives of this study were (1) to compare predictive parameters with other models, including Hestia criteria, RIETE model, PESI, simplified PESI, Spanish score, and Geneva score (5,22,25,29,30,35).*

We have briefly described the basic details and weighting method of these models in the variables section:

*The other five models - Hestia criteria, RIETE model, PESI, simplified PESI, Spanish model, and Geneva score - were evaluated based on their original descriptions and validated cut-off points. Each prognostic category was assigned a 30-day mortality probability value. Predicted and observed deaths at 30 days were compared to assess the statistical validity of the models.*

In the statistics section, we have provided a detailed account of the predictive parameters evaluated in this model comparison:

*As measures of performance, sensitivity, specificity, positive and negative predictive values (PPV and NPV), and positive and negative likelihood ratios (PLR and NLR) were estimated.*

Since the comparison of the EPIPHANY index with other models is a secondary objective, the results have been incorporated into Appendix Table 3.

- Methods section: the outcome were complications or serious complications? This point is critical. Moreover, I did not find in the Methods sections a definition of complications or serious complications.

Based on this suggestion, we have provided clarification that the complications evaluated as part of the primary endpoint refer specifically to serious complications. The variables section now includes a clear and detailed description of this endpoint in the following paragraph, while a detailed description of the serious complications found in the patients is presented in Table 2:

*In our model validation process, we selected serious complications within 15 days as the primary endpoint. This choice was made based on its better fit with the clinical scenario of managing PE in cancer patients as outpatients. This decision was previously supported in the literature [8,14]. This endpoint includes the development of any of the following events: hypotension (systolic blood pressure <90 mmHg), acute respiratory insufficiency, fibrinolysis, major bleeding (intracranial, intraspinal, intraocular, retroperitoneal, or pericardial, associated with decreased hemoglobin by at least 2 g/dl or requiring the transfusion of two units of red blood cells), acute heart failure secondary to right ventricle failure, acute kidney failure, admission into the Intensive Care Unit, need of cardiopulmonary resuscitation, non-invasive mechanical ventilation, oro-tracheal intubation or death. To be deemed outcomes, these events must have occurred after the objective diagnosis of PE. Should they have occurred before then (e.g., at the time of debut), the events were coded as predictors.*

Results section

- Please use lung cancer instead of broncho-pulmonary cancer.

We have changed the term throughout the text in accordance with the reviewer's recommendation.

- Of note, 12.3% of the sample (110 patients) had PE during anticoagulant treatment. Patients with cancer that develop PE during anticoagulant treatment should not be included in the analyses. This population differ too much with patients with cancer that develop PE without anticoagulant treatment.

We agree that it may be a relevant issue to consider. In response to this query, we have evaluated in more detail the profile of patients who were receiving previous anticoagulation therapy. We have conducted a sensitivity analysis by excluding the 110 patients who had received anticoagulation therapy prior to their PE diagnosis. The analysis demonstrated that the prior use of anticoagulant agents did not appear to modify the severity of PE, with a complication rate of 107 (11.8%), 93 (11.8%), and 14 (12.6%) in the overall cohort, those without and with previous anticoagulation therapy, respectively. We have reported these data in the revised version of the manuscript. Additionally, we performed a sensitivity analysis by reconstructing the EPIPHANY index excluding these 110 subjects, and found that the results were very similar. Therefore, we have included this analysis in the manuscript as a sensitivity analysis:

*No substantial changes were observed in the performance of the index after excluding individuals who had received anticoagulants before the PE, as shown in Annex Figure 1B.*

We have elected to report the results for the entire cohort, as there seems to be no significant difference in including or excluding patients with prior anticoagulation therapy. The analysis for patients without prior anticoagulation therapy is presented as a sensitivity analysis. However, we acknowledge the reviewer's concerns regarding the potential impact of this variable on the model's performance, particularly in cases where supratherapeutic doses of heparin are necessary for retrombosis. To address this concern, we have added a clarification in the discussion section to alert readers to this variable and its potential influence on the model's performance. We have commented this as a limitation:

*It is important to acknowledge the potential limitations of our study, specifically the possible impact of prior anticoagulant use on the performance of the model. This is especially relevant in cases where supratherapeutic doses of heparin need to be administered for retrombosis. As such, we caution readers to consider this variable when interpreting our results and suggest that further research is necessary to determine the true impact of this variable on the performance of the model.*

Table 2 should be revisited.

- File of any complication need to be the sum of the other files

We have thoroughly reviewed Table 2 and found no obvious issues with the count. It is important to note that the complications are not mutually exclusive. Additionally, the table stratifies patients based on whether the management was done in the hospital or at home, with the total sum reflected in the right-hand column. Moreover, the percentages in the rows reflect different denominators, and thus, the sum of these percentages does not correspond to the percentage of the total row, due to mathematical reasons. We have clarified in the method and in the footnote of Table 2 that the categories are not mutually exclusive.

- define other serious complications due to is critical to define the outcome.

We have included this information in the footnote of table 2 and clarified the definition of endpoints. The category “other complications” was created to encompass any circumstances that the investigators perceived as serious but did not fit into any specific category. Out of the 900 patients in the series, 18 (2%) experienced such complications, including acute intestinal ischemia (n=1), need for thrombectomy (n=1), severe pain requiring intravenous opioids (n=1), severe pleural effusion requiring thoracentesis (n=1), severe anemia (Hb 6 mg/dL) (n=1), pulmonary infarction and bronchopleural fistula (n=1), intestinal perforation (n=1), acute pancreatitis (n=1), grade 4 thrombocytopenia due to chemotherapy (n=1), severe pulmonary hypertension (n=1), ischemic stroke (n=2), bronchoaspiration (n=1), hemothorax (n=1), cardiac tamponade (n=1), severe infection (n=2), and other serious complications, not specified (n=1).

We have added this clarification of the other complications as a footnote to table 2.

- about RV failure. It is different from RV dysfunction? Please make a definition

We have revised the terminology in accordance with the reviewer's suggestion, using the term "right ventricular dysfunction" and providing an appropriate reference.

- About Hypotension: This complication can be at the moment of the PE event and for that reason can be a marker of the severity more than a complication during hospitalization. The same for thrombolytic treatment.

We appreciate this excellent point and agree that we need to provide further clarification on this issue. The goal of this model is to create a decision tree that aligns with the normal evaluation and management flow of these patients. Thus, when a patient arrives at the Emergency Department, the first evaluation is to assess vital signs and determine whether the patient with suspected PE has an unstable clinical and hemodynamic status. This is explicitly reflected in the HESTIA criteria, at the top of the decision tree. The most critical component of validating this model has been determining, with controlled precision (error <2%), the rate of complications in apparently stable individuals defined by the absence of any of the events that define the Hestia criteria. The emphasis on this result is due to the fact that the Hestia criteria have been proposed as a selection criterion for outpatient management^[10]^. The hypothesis is that if patients are incorrectly classified, their prognosis may worsen, especially if they are managed at home reducing the level of support. Therefore, the criterion followed here has been to try to mimic this natural flow of clinical information, distinguishing first between those subjects who had or did not have the events defined in Hestia. Therefore, in cases where a severe event was present prior to the objective diagnosis of PE, the criterion employed was to classify it as a defining predictor of high risk according to the Hestia criteria. Alternatively, in cases where a severe event was not present at the time of PE diagnosis but developed later, the patient was classified as not initially meeting Hestia criteria but experiencing an endpoint subsequently. All of this was then qualified with the rest of the model's variables. The reason for this approach was to determine the ability of Hestia criteria to predict subsequent severe complications based on the patient's baseline clinical status, whether stable or unstable.

In any case, in agreement with the reviewer, we have briefly explained this criterion in the methods section:

*To be deemed outcomes, these events must have occurred after the objective diagnosis of PE. Should they have occurred before then (e.g., at the time of debut), the events were coded as predictors. The reason for this approach was to rapidly classify patients based on the initial Hestia criteria and specifically investigate their ability to predict the eventual development of severe complications after the objective diagnosis of PE, based on the baseline clinical presentation.*

Reviewer #2: Using a prospective approach, the authors evaluated serious complications of pulmonary embolisms among cancer patients and validated EPIPHANY index in this cohort. In this multi-institution study, 900 patients were recruited, and the rates of serious complications were reported. Further, the EPIPHANY Index was examined for these patients along with the Hestia criteria and their usefulness was assessed. The study is well-designed. However, there are a few issues that need to be clarified:

Firstly, we would like to express our gratitude to Reviewer #2 for carefully reviewing our manuscript, recognizing it as a well-designed study, and proposing ways to further strengthen the article.

1. Introduction (Page 6: “Nonetheless, outpatient management of cancer-related PE is not based on quality evidence, and there is a glaring dearth of prospective data”): there is a good consensus in the guidelines regarding outpatient management of PE in cancer patients (https://doi.org/10.1016/S1470-2045(19)30336-5; https://doi.org/10.1200/JCO.19.01461; https://doi.org/10.6004/jnccn.2018.0084). I suggest the authors change this statement.

We have revised this sentence in accordance with the suggestion of reviewer #2, including recent references to the NCCN 2022 and ASH 2020 guidelines addressing this topic. However, we believe that outpatient management of cancer-related PE still requires further research before it can be considered fully standard. For example, the ASH 2020 clinical practice guidelines reviewed the literature and stated that several studies have demonstrated the effective and safe treatment of low-risk PE patients at home. However, the quality of evidence supporting this recommendation was considered of very low certainty, making it a conditional recommendation. The ASH 2020 guideline panel suggests *offering home treatment over hospital treatment (conditional recommendation based on very low certainty in the evidence of effects* ⨁○○○)^[11]^.

The NCCN 2022 guideline cautiously supports outpatient management of low-risk PE ^[12]^: "*Since the 3-month mortality rate of patients with PE has been reported to be 15%, outpatient management should be limited to individuals at low risk as identified by clinical, laboratory, and imaging assessment*." Therefore, in accordance with the reviewer's recommendation, we have modified this sentence as follows:

"*Although there are some limited data and guidelines suggesting that outpatient management of cancer-related PE is feasible, high-quality prospective data supporting this approach is still lacking (3, 19-21).*"

2. Introduction (Page 6: “or the like”): Please list the rules that the authors are referring one.

We have modified this sentence by adding the appropriate references in the text:

*To date, clinical trials of home management have selected participants using decision rules, such as the Hestia criteria or adaptations carried out by other authors [3,19], contraindicating lowering the level of support in patients with any exclusion criterion [19,22,23].*

3. Introduction (Page 7: “The EPIPHANY Index was designed as a modified extension of the Hestia clinical decision rule to pragmatically select patients with low-risk, cancer-related PE [26].”). Please make sure the reference is correct to this statement.

Thank you for pointing out this error. The reference was indeed incorrect. In the updated version of the manuscript, we have used the correct one.

4. Inclusion criteria included active solid neoplasm. This needs to be stated in the title and the abstract, that the study is on cancer patients with solid tumors.

According to the recommendation of reviewer #2, we have adapted the abstract to clarify that the patients had active cancer or were receiving antineoplastic therapy.

We also updated the title as suggested:

*Prediction of serious complications in patients with pulmonary thromboembolism and solid cancer: validation of the EPIPHANY Index in a prospective cohort of patients from the PERSEO Study*

5. What is the timeframe for the “Anticoagulation therapy at diagnosis of PE”? Why did only 12.3% got anticoagulants at the time of diagnosis?

The expression used in the table was actually confusing. It referred to the fact that 12.3% of individuals had been exposed to anticoagulation therapy before the PE episode (e.g., primary thromboprophylaxis). We previously discussed the impact of this subgroup on the overall performance of the model with reviewer #1 (see above) and introduced a sensitivity analysis. It is important to note that all patients received anticoagulation therapy after the diagnosis of PE.

To clarify this matter, we have updated the language in Table 1 to state "Anticoagulation therapy prior to the diagnosis of PE (e.g., primary thromboprophylaxis)".

6. In table 1, please separate the type of PE. Maybe to Laterality, location, and presentation.

Although we feel that Table 1 adequately displays the most important data, we have addressed this query by generating an additional table that breaks down the baseline characteristics according to the location of the PE (central or peripheral) and the presentation (suspected or unsuspected). This information has been included as Supplementary Table 1.

7. The type of care doesn’t add up to 900. What is the remaining group?

We appreciate the reviewer #2 for identifying the typographical error. Indeed, 549 patients were managed in the hospital, not 540 as previously stated. We have corrected the figure in the revised version of the document.

8. Some complications in Table 2 are not defined in the methods, please clarify if only the outcomes mentioned in the methods are considered serious complications within 15 days and were used in the validation step. If that’s the case, only keep these complications in table 2 and the remaining complications can be summarized in a supplemental table.

Thank you for bringing this to our attention. We have thoroughly checked the description of all severe complications in Table 2 against our methodology and have ensured that they are all adequately described.

9. Please define the abbreviations in the manuscript on their first occurrence. Abbreviations in the tables need to be listed in the footnote of the tables as well.

We have ensured that all acronyms used in the manuscript are properly defined in their first appearance, as suggested by the reviewer. We have also added the explained abbreviations at the bottom of the tables.

10. Please use the full-scale for the y-axis in Annex figure 3

As per reviewer #2's suggestion, we have modified the plot to include the full range on both axes.

1. Jiménez, David, et al. "Simplification of the pulmonary embolism severity index for prognostication in patients with acute symptomatic pulmonary embolism." Archives of internal medicine 170.15 (2010): 1383-1389.
2. Weeda ER, Caranfa JT, Zeichner SB, Coleman CI, Nguyen E, Kohn CG. External validation of generic and cancer-specific risk stratification tools in patients with pulmonary embolism and active cancer. J. Natl. Compr. Cancer Netw. Harborside Press, LLC; 2017;15:1476–82.
3. Font C, Carmona-Bayonas A, Fernández-Martinez A, Beato C, Vargas A, Gascon P, et al. Outpatient management of pulmonary embolism in cancer: data on a prospective cohort of 138 consecutive patients. J. Natl. Compr. Canc. Netw. 2014;12.
4. Kline JA, Roy PM, Than MP. Derivation and validation of a multivariate model to predict mortality from pulmonary embolism with cancer: The POMPE-C tool. Thromb Res. 2012;199.
5. Den Exter PLPL, Gómez V, Jiménez D, Trujillo-Santos J, Muriel A, Huisman M V, et al. A clinical prognostic model for the identification of low-risk patients with acute symptomatic pulmonary embolism and active cancer. Chest [Internet]. 2013;143:138–45. Available from: http://www.ncbi.nlm.nih.gov/pubmed/22814859
6. Ahn S, Lee Y-S, Kim WY, Lim KS, Lee J-L. Prognostic value of treatment setting in patients with cancer having pulmonary embolism: comparison with the Pulmonary Embolism Severity Index. Clin. Appl. Thromb. SAGE Publications Sage CA: Los Angeles, CA; 2017;23:615–21.
7. Li X, Hu Y, Lin P, Zhang J, Tang Y, Yi Q, et al. Comparison of different clinical prognostic scores in patients with pulmonary embolism and active cancer. Thromb. Haemost. Georg Thieme Verlag KG; 2021;121:834–44.
8. Carmona-Bayonas, A., Font, C., Jiménez-Fonseca, P., Fenoy, F., Otero, R., Beato, C., ... & Vicente, V. (2016). On the necessity of new decision-making methods for cancer-associated, symptomatic, pulmonary embolism. Thrombosis research, 143, 76-85.
9. Carmona-Bayonas, A., Jiménez-Fonseca, P., Font, C., Fenoy, F., Otero, R., Beato, C., ... & Vicente, V. (2017). Predicting serious complications in patients with cancer and pulmonary embolism using decision tree modelling: the EPIPHANY Index. British journal of cancer, 116(8), 994-1001.
10. Roy, P. M., A. Penaloza, and O. Hugli. "Hestia rule versus simplified PESI for home treatment of patients with acute pulmonary embolism: a multinational randomised clinical trial (HOME‐PE)." *Eur Heart J* 42.33 (2021): 3146-3157.
11. Ortel, Thomas L, et al. "American Society of Hematology 2020 guidelines for management of venous thromboembolism: treatment of deep vein thrombosis and pulmonary embolism." *Blood advances* 4.19 (2020): 4693-4738.
12. Streiff MB, Ashrani AA, Elshoury A, et al. Cancer-Associated Venous Thromboembolic Disease. NCCN Clinical Practice Guidelines in Oncology (NCCN Guidelines®) v1.2022. 2022.
